# Supplementary material for: Do protein language models learn phylogeny?
Source: Brief Bioinform. 2025 Feb 23;26(1):bbaf047. doi: 10.1093/bib/bbaf047 (PMC11847157; doi:10.1093/bib/bbaf047)
Supplement: PlmEvo_supplmentary_revised [file plmevo_supplmentary_revised.pdf]

Supplementary material

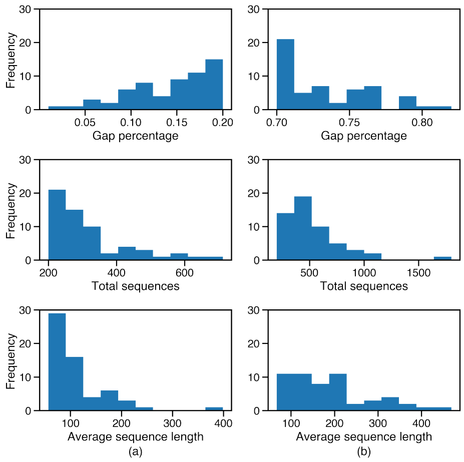

Fig. S1: Histogram plot showing the distribution of gap percentage, number and lengths of sequences in MSAs for low-gap (a) and high-gap (b) datasets.

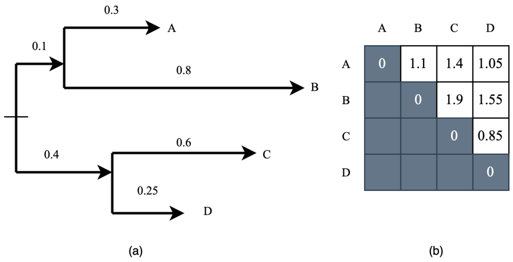

Fig. S2: Figure shows an example phylogenetic tree with four sequences - A, B, C and D (1) and corresponding LG matrix (2).

Table S1: Pfam datasets used for analysis.

**Family low-gap**	**Domain low-gap**	**Family high-gap**	**Domain high-gap**
PF04298	PF00158	PF01055	PF04205
PF07907	PF09278	PF12686	PF04427
PF02702	PF12002	PF20060	PF13508
PF04961	PF12775	PF03914	PF04264
PF04461	PF17941	PF02586	PF05192
PF04403	PF16658	PF02365	PF01156
PF01668	PF01706	PF01139	PF11799
PF01450	PF02261	PF00617	PF03796
PF04304	PF14278	PF03150	PF01288
PF03975	PF09361	PF01297	PF09994
PF05635	PF18565	PF20415	PF13280
PF19609	PF17146	PF00856	PF16124
PF03862	PF01948	PF00902	PF13356
PF01219	PF00707	PF02104	PF17852
PF06421	PF06071	PF02811	PF12804
PF14842	PF10437	PF12276	PF01510
PF03788	PF13667	PF01368	PF04122
PF00934	PF00189	PF02383	PF01369
PF02049	PF11760	PF04051	PF01302
PF02617	PF02650	PF12704	PF01266
PF04341	PF10369	PF02517	PF00006
PF01052	PF01037	PF01728	PF12697
PF02700	PF06130	PF01885	PF00557
PF14841	PF02594	PF00636	PF01388
PF00986	PF07554	PF01196	PF05257
PF08439	PF09269	PF13423	PF05226
PF02805	PF00366	PF01926	PF01149
PF01313	PF03719		
PF00831	PF03880		
PF02073	PF20554		

Table S2: Total under performing datasets (where $ESS_\rho \leq 0.3$, $ESS_r \leq 0.3$)

**Name**	**low-gap**	**high-gap**
ESM2-embed	5, 5	7, 3
PT-embed	1, 1	7, 5
MSA-embed	0, 0	28, 28
MSA-col1	1, 1	38, 46

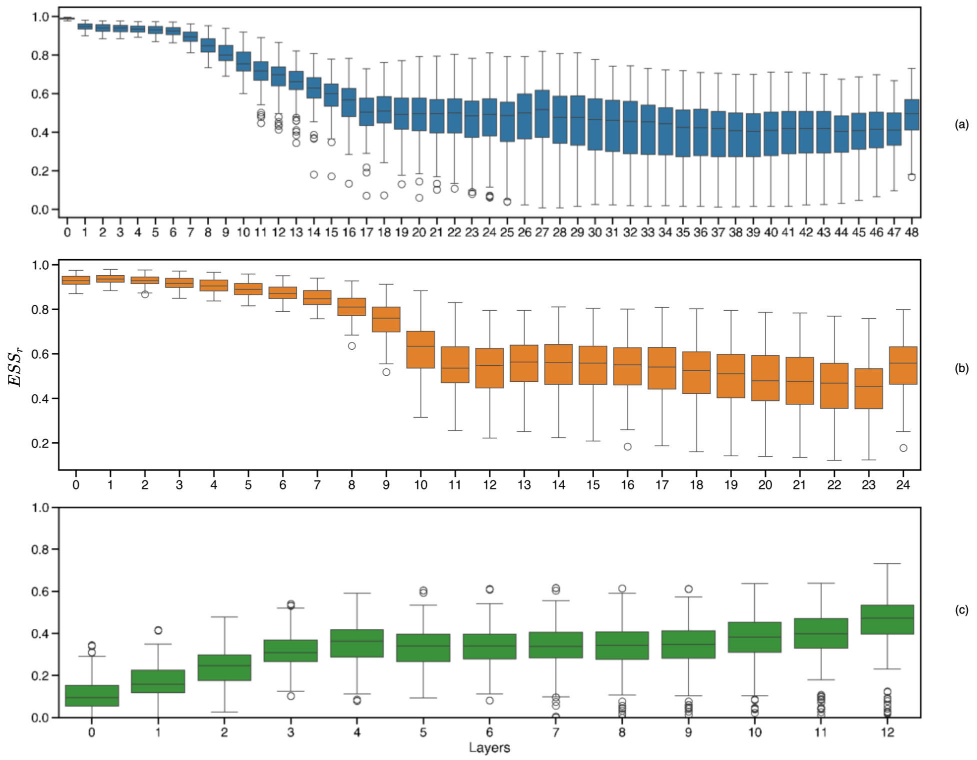

Fig. S3: Evolutionary similarity score (ESS_r; y-axis) between layer-specific pLM matrix (x-axis) and onehot matrix. Boxplots are based on 114 Pfam datasets for ESM2-embed (a) and PT-embed (b). In case of MSA-Transformer (c), boxplots are based on 100 Pfam entries.

Table S3: Difference in the absolute ESS between MSA-col2 and MSA-col1. Mean and standard deviation are shown.

**Dataset category**	ESS_r	ESS_ρ
lowgap	0.003 (0.16)	0.004 (0.12)
highgap	0.28 (0.19)	0.19 (0.17)

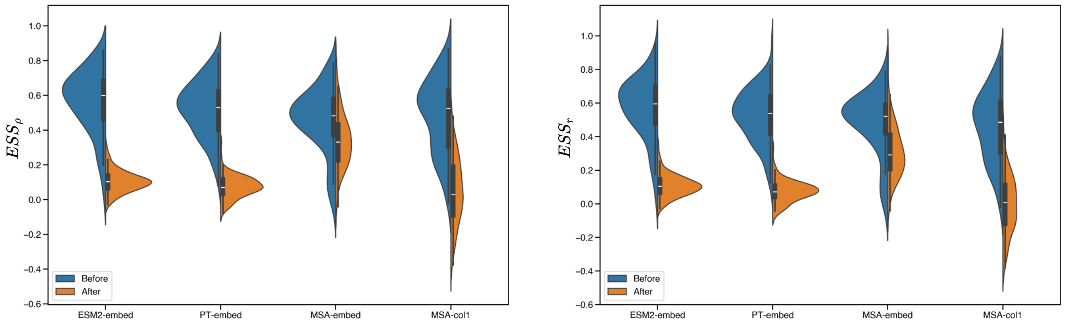

(a) (b)

Fig. S4: Distributions of ESS_ρ (a) and ESS_r (b) before and after shuffling of amino acids in the sequences. Sequence are shuffled for 80% of the total non-aligned sequences for ESM2-embed and PT-embed. For MSA-embed and MSA-col1, we shuffle on aligned sequences.

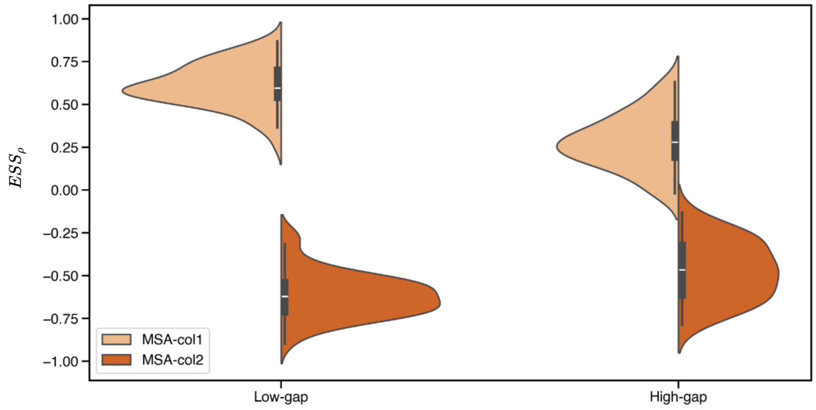

Fig. S5: Distributions of ESS_ρ of select column attention heads in MSA-Transformer, across all applicable low-gap and high-gap datasets; the scores for MSA-col2 (layer three, head 12; dark orange) negatively mirrors MSA-col1 (layer 1, head 5; light orange).

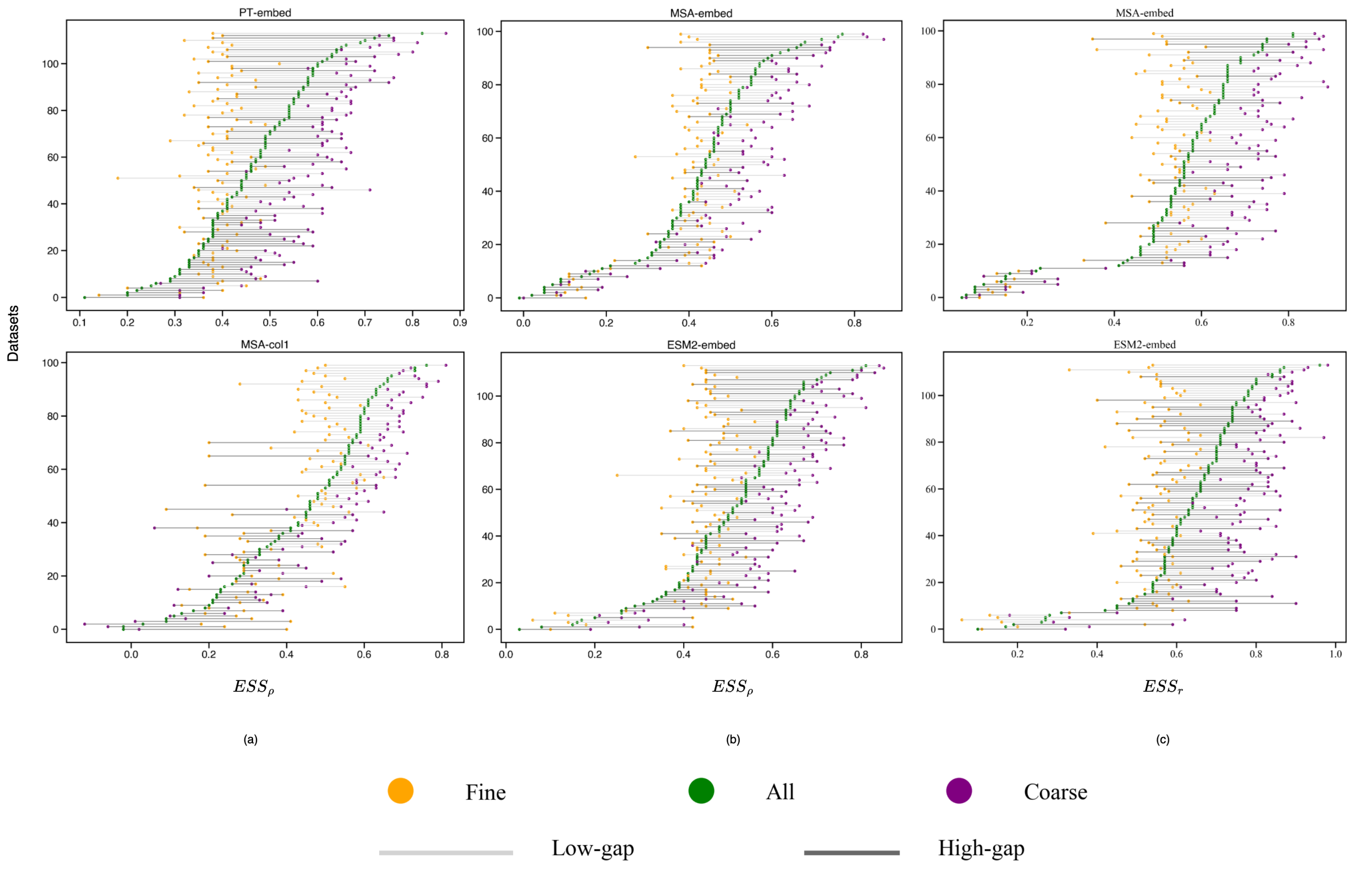

Fig. S6: Dot plot representing the "fine" (yellow) and "broad" (purple) ESS_ρ (a, b) and ESS_r (c) (shown on x-axis), as well as the ESS including "all" (pairs of) sequences (green), for all Pfam datasets sorted on the y-axis in ascending order (top-to-bottom) by the "all" correlation. (a) We include LG matrix vs. PT-embed (top panel) and LG matrix vs. MSA-col1 (bottom panel). (b) We include LG matrix vs. MSA-embed (top panel) and ESM2 matrix vs. MSA-col1 (bottom panel). (c) We include LG matrix vs. MSA-embed (top panel) and LG matrix vs. ESM2-embed (bottom panel). Lines in light grey represent low-gap and dark grey represent high-gap Pfam datasets.

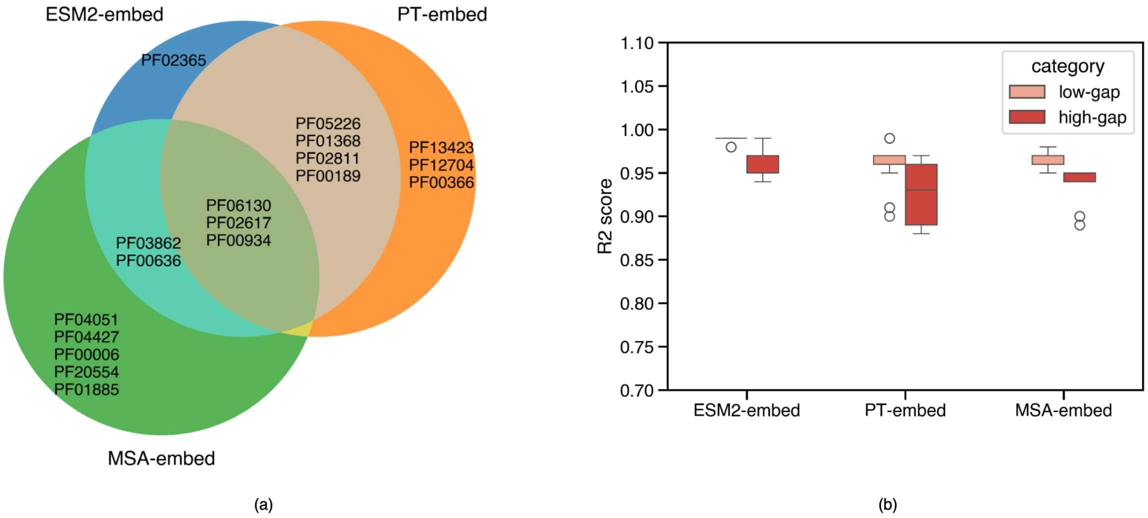

Fig. S7: A) Venn diagram showing datasets used to train elastic net regression for each pLM representation. B) Boxplot showing the distribution of R^2 score on test sets for each pLM representation. The score distibution is shown for both "gap" categories. .

Table S4: ESS_r using salient and bottom neurons for ESM2-embed

**Dataset**	**All**	**Top**				**Bottom**
		**(10%)**	**(25%)**	**(50%)**	**(75%)**	**(25%)**
PF03862	0.84	0.95	0.95	0.93	0.9	0.81
PF00189	0.86	0.94	0.94	0.92	0.89	0.83
PF02617	0.87	0.96	0.96	0.93	0.9	0.85
PF00934	0.89	0.91	0.92	0.92	0.9	0.88
PF06130	0.95	0.98	0.98	0.98	0.97	0.94
PF02365	0.73	0.75	0.76	0.74	0.74	0.71
PF00636	0.73	0.85	0.84	0.81	0.78	0.71
PF01368	0.73	0.84	0.83	0.79	0.77	0.72
PF05226	0.79	0.8	0.81	0.8	0.8	0.78
PF02811	0.83	0.95	0.92	0.88	0.86	0.81

Table S5: ESS_r using salient and bottom neurons for PT-embed

**Dataset**	**All**	**Top**				**Bottom**
		**(10%)**	**(25%)**	**(50%)**	**(75%)**	**(25%)**
PF00189	0.79	0.84	0.84	0.83	0.82	0.75
PF00366	0.80	0.79	0.82	0.83	0.82	0.79
PF02617	0.82	0.86	0.88	0.86	0.83	0.79
PF00934	0.88	0.85	0.88	0.88	0.88	0.85
PF06130	0.97	0.98	0.98	0.98	0.98	0.93
PF12704	0.68	0.69	0.71	0.7	0.7	0.66
PF13423	0.73	0.8	0.77	0.75	0.75	0.72
PF05226	0.76	0.77	0.8	0.78	0.78	0.74
PF01368	0.76	0.78	0.82	0.82	0.79	0.72
PF02811	0.86	0.91	0.89	0.89	0.89	0.81

Table S6: ESS_r using salient and bottom neurons for MSA-embed

**Dataset**	**All**	**Top**				**Bottom**
		**(10%)**	**(25%)**	**(50%)**	**(75%)**	**(25%)**
PF20554	0.73	0.8	0.79	0.79	0.76	0.7
PF02617	0.74	0.89	0.85	0.81	0.78	0.69
PF00934	0.78	0.82	0.84	0.83	0.82	0.75
PF03862	0.78	0.94	0.89	0.9	0.86	0.72
PF06130	0.79	0.93	0.93	0.91	0.88	0.72
PF00636	0.65	0.63	0.67	0.68	0.68	0.6
PF04427	0.69	0.79	0.8	0.76	0.71	0.66
PF01885	0.71	0.79	0.82	0.8	0.76	0.67
PF04051	0.72	0.82	0.83	0.8	0.77	0.66
PF00006	0.72	0.81	0.85	0.81	0.78	0.69

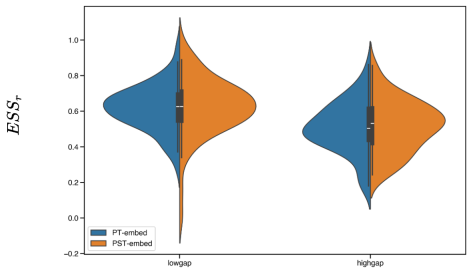

(a)

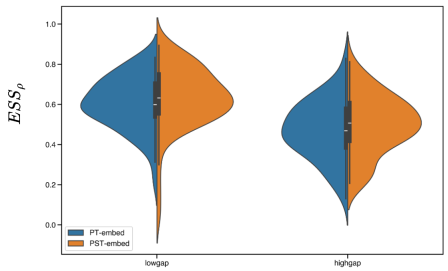

(b)

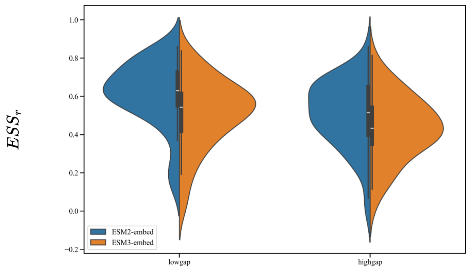

(c)

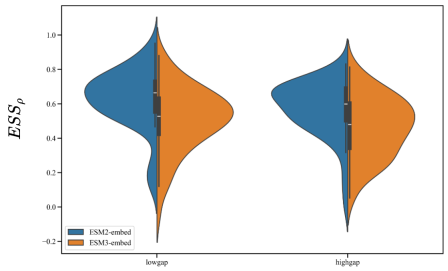

(d)

Fig. S8: Distributions of ESS_r of PT-embed vs PST-embed (a) and ESM2-embed vs ESM3-embed (b) across all applicable low-gap and high-gap datasets. Distributions of ESS_ρ of PT-embed vs PST-embed (c) and ESM2-embed vs ESM3-embed (d) across all applicable low-gap and high-gap datasets.

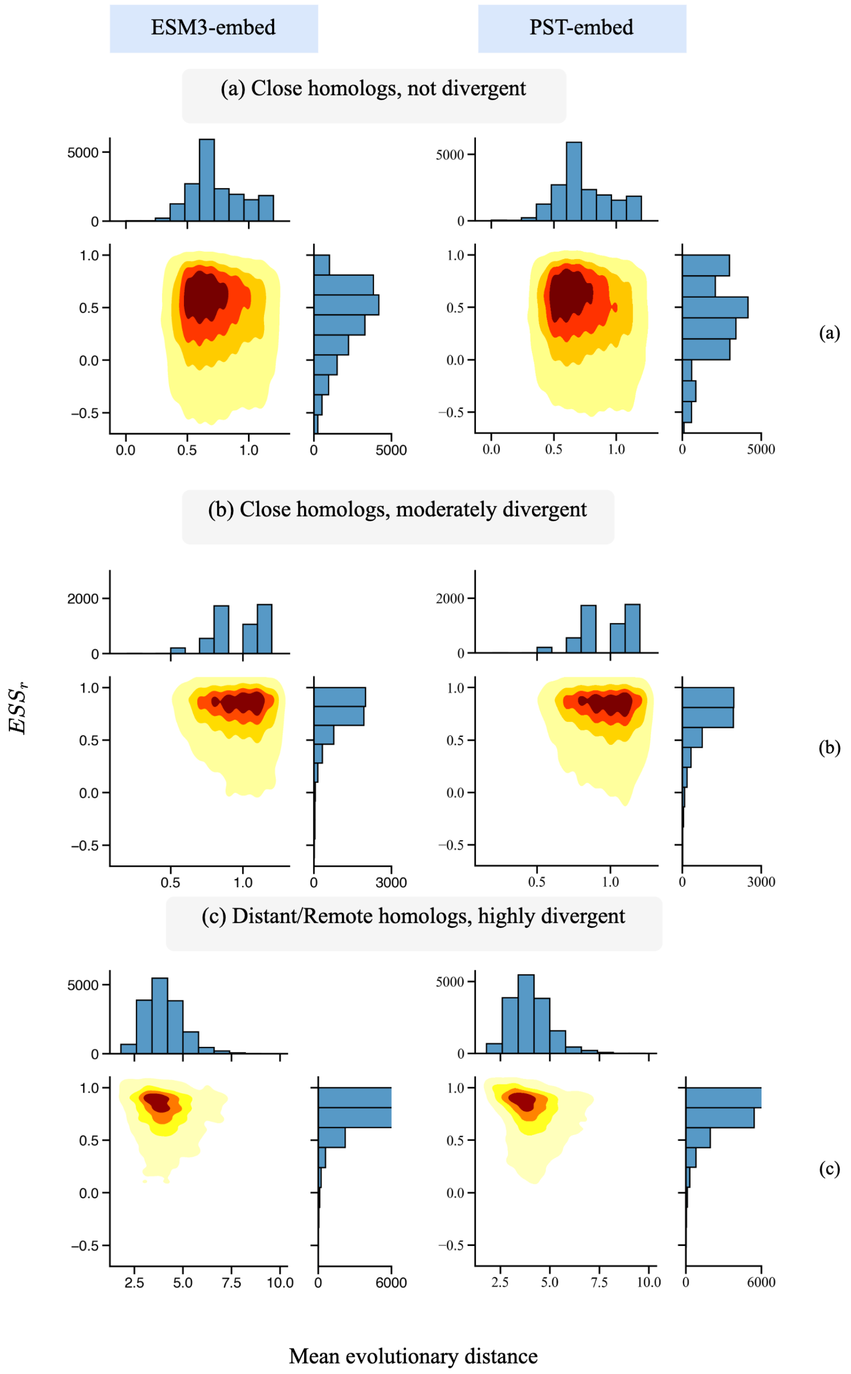

Fig. S9: Analysis of ESS_r for different evolutionary distances for ESM3-embed and PST-embed. Density of ESS_r for "close homologs, not divergent" (a), "close homologs, moderately divergent" (b) and "distant/remote homologs, highly divergent" (c) relative to mean evolutionary distance in each group (from reference sequence).

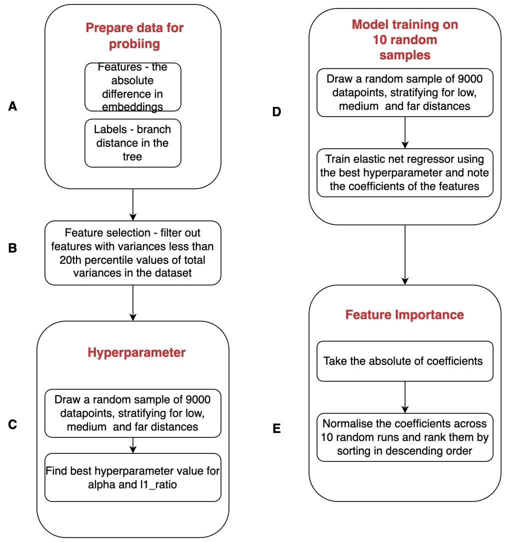

Fig. S10: Evolutionary probe workflow. (a) Data preparation. (b) Feature selection. (c) Hyperparameter selection. (d) Model training. (e) Feature importance.
